# The Cellular and Mitochondrial Consequences of Mevalonate Pathway Inhibition by Nitrogen-Containing Bisphosphonates: A Narrative Review

**DOI:** 10.3390/ph18071029

**Published:** 2025-07-11

**Authors:** Adrianna Budzinska, Wieslawa Jarmuszkiewicz

**Affiliations:** Laboratory of Mitochondrial Biochemistry, Department of Bioenergetics, Institute of Molecular Biology and Biotechnology, Adam Mickiewicz University, Collegium Biologicum, Uniwersytetu Poznanskiego 6, 61-614 Poznan, Poland; adrianna.budzinska@amu.edu.pl

**Keywords:** mevalonate pathway, mitochondria, nitrogen-containing bisphosphonates

## Abstract

Nitrogen-containing bisphosphonates (N-BPs) are commonly used drugs in the treatment of bone diseases due to their potent inhibition of the mevalonate pathway, leading to disrupted protein prenylation and reduced osteoclast activity. Although N-BPs are effective in reducing bone resorption, increasing evidence indicates their side effects on various non-skeletal cells. The aim of this review is to synthesize the current knowledge on the cellular and molecular effects of N-BPs outside the skeletal system, with particular emphasis on their impact on mitochondrial function and energy metabolism. At the cellular level, N-BPs may reduce viability, modulate inflammatory responses, trigger apoptosis, disrupt cytoskeletal organization, and influence signaling and energy metabolism. N-BPs may also impair the prenylation of proteins essential for mitochondrial dynamics and quality control, and may disrupt Ca^2+^ homeostasis. As we have shown in endothelial cells, by inhibiting the mevalonate pathway, N-BPs may lead to a reduction in key components of the mitochondrial respiratory chain, such as coenzyme Q (CoQ) and *a*-heme. These effects can contribute to impaired mitochondrial respiratory function, increased oxidative stress, and mitochondria-dependent apoptosis, affecting cellular energy metabolism and viability. These findings underscore the multifaceted impact of N-BPs beyond bone, emphasizing the importance of mitochondrial health and energy metabolism in understanding their broader biological effects and potential adverse outcomes.

## 1. Introduction

### 1.1. History of Bisphosphonates

Osteoporosis is the most common metabolic bone disease, particularly prevalent among postmenopausal women. As the world’s population ages, the incidence of osteoporosis is increasing, with over 200 million people now suffering from the disease [1]. Bone health is maintained by a delicate balance between bone resorption by osteoclasts and bone formation by osteoblasts. This balance can be disturbed by many factors, including aging, genetic disease, lifestyle and endocrine disorders [2]. The primary goal of osteoporosis treatment is the prevention of fractures, which can be achieved either by stimulating bone formation or, more commonly, by inhibiting bone resorption. Bisphosphonates (BPs) are the most commonly prescribed antiresorptive drugs, approved as first-line treatment for osteoporosis, Paget’s disease and bone diseases in cancer patients [3]. Their clinical use has also been extended to the treatment of bone metastases and other diseases associated with increased bone turnover.

**Figure 1:**
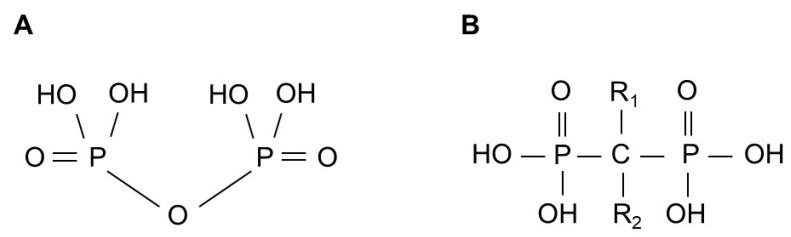
General structure of pyrophosphate (**A**) and bisphosphonate (**B**).

BPs are stable synthetic pyrophosphate analogs, in which the central carbon atom replaces the oxygen bridge and carries two variable side chains, R_1_ and R_2_ (Figure 1). Historically, before their potential in preventing bone resorption was discovered in 1969 [4,5], they were first used industrially for their calcium-chelating properties [6,7] as water softeners, corrosion inhibitors and fertilizers. Manipulation of BP side chains has led to the discovery of new compounds with different potencies, while the synthesis of BPs containing a nitrogen atom in the R2 alkyl chain has created a new type of drugs with significantly improved antiresorptive properties [8].

### 1.2. Types of BPs

BPs are divided into two main groups based on their R_2_ side chain—non-nitrogen-containing bisphosphonates and nitrogen-containing bisphosphonates (N-BPs) [9]. N-BPs can be further divided into second-generation (including alendronate) and more efficient third-generation (including ibandronate and zoledronate) drugs. First-generation BPs—non-nitrogen-containing compounds such as etidronate and clodronate—can interfere with adenosine triphosphate (ATP)-dependent enzymes by generating non-hydrolyzable ATP analogs [10]. These analogs compete with ATP, ultimately inducing apoptosis in osteoclasts. Most often, these types of BPs contain a simple hydroxyl group in the R1 chain, while the R2 chain may contain a chlorine atom (in clodronate) or an alkyl group (in etidronate). Clodronate is metabolized by class II aminoacyl-tRNA synthases to adenosine-5′-(β,γ-dichloromethylene) triphosphate (AppCCl_2_p), a toxic ATP analog that accumulates and disrupts cellular energetics and other cellular processes [11,12]. Non-hydrolyzable ATP analogs may reduce cytokine release, offering potential in treating inflammatory conditions. They reach mitochondria, changing oxygen consumption and depolarizing the mitochondrial membrane potential [13].

In 1998, Luckmann et al. suggested that N-BPs induce apoptosis in osteoclasts and inhibit bone resorption by disrupting protein prenylation, likely through inhibition of mevalonate pathway enzymes [14]. Indeed, in contrast to non-nitrogen-containing BPs, N-BPs act by inhibiting farnesyl diphosphate synthase (FPP synthase) in the mevalonate pathway, preventing prenylation of proteins essential for osteoclast survival and function (e.g., membrane ruffling and stress fiber assembly) [13,14,15]. The greater efficacy of N-BPs compared to first-generation BPs is attributed to their greater affinity for bone mineral [15]. The bone-targeting properties of N-BPs provide selective accumulation at resorption sites, where they effectively inhibit osteoclast recruitment, activity and induce apoptosis. The most commonly used N-BPs are alendronate, prescribed primarily to prevent bone fractures, and zoledronate, a more potent agent used to prevent pathological fractures or treat bone loss associated with cancer [16].

### 1.3. Side Effects of N-BPs

N-BPs can be administered intravenously or orally. However, oral administration is linked with poor absorption and has many contradictions. The negative charge of N-BPs impedes their transport across the lipophilic membrane. A similar effect is achieved by complexing with calcium ions, to which they owe their high affinity to sites of intense bone resorption. Most of the contradictions of N-BP use are connected to the frequent comorbidities of osteoporosis, e.g., inflammatory bowel diseases (Crohn’s disease, ulcerative colitis, celiac disease), small bowel resection or gastojejunostomies [17]. Intravenous treatment with N-BPs, particularly zoledronate, shows greater drug adhesion to the bone surface, with approximately 50–70% of the dose binding to bone minerals within 6–10 h. Compared to the low bioavailability (~5%) of oral administration, intravenous treatment demonstrates its superiority [18,19].

The use of alendronate, a second-generation bisphosphonate, has tripled from 2.7% in the 1990s to 7.8% in less than a decade [20]. The global market of N-BPs continues to grow. Given this, the side effects of these medications have been extensively studied (Table 1). Upper gastrointestinal side effects are most commonly reported with oral N-BPs. Failure to maintain an upright position for 60 min after taking the drug caused esophagitis in patients [21].

The most feared side effect of intravenous N-BPs is osteonecrosis of the jaw, which is likely due to the antiangiogenic properties of N-BPs. Bisphosphonate-related osteonecrosis of the jaw (BRONJ) is thought to be related to the use of high doses of the drugs, but the condition also affects patients taking lower doses. More recently, it has been proposed that BRONJ may be caused by the presence of Actinomyces infection, decreased bone turnover, inhibition of angiogenesis and immune dysregulation [23]. Ziebart et al. suggested that the effect of N-BPs on the onset of BRONJ could be reversed [24]. By studying the products of the mevalonate pathway, they found that one of them, geranylgeraniol (GGOH), when added to in vitro cultures of HUVEC cells, reversed the effects of clodronate, zoledronate, pamidronate and ibandronate.

For a more comprehensive overview of N-BP pharmacology, their mechanisms of action in bone, and known extraskeletal side effects, readers are encouraged to consult several in-depth reviews on these topics [5,9,21,25,26].

## 2. Mevalonate Pathway—A Biological Role and Products

**Figure 2:**
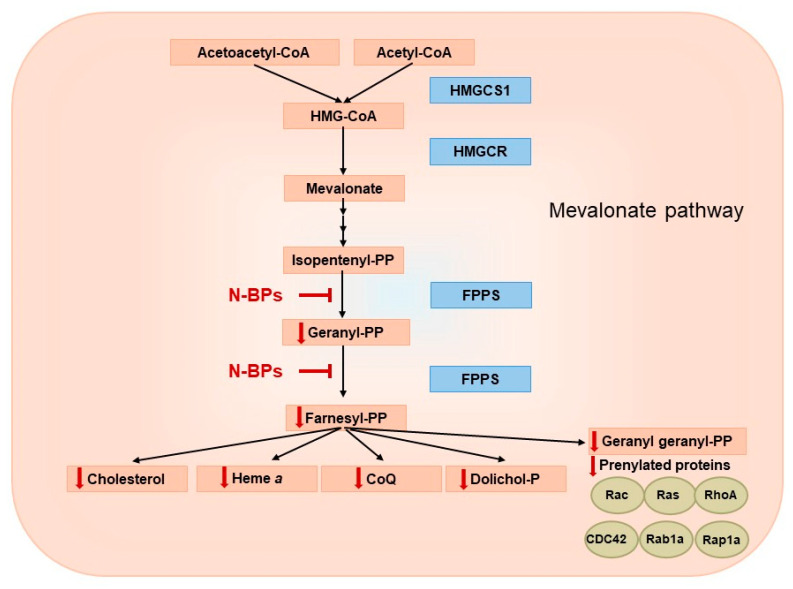
Potential reduction in mevalonate pathway products via farnesyl diphosphate synthase (FPPS) inhibition by nitrogen-containing bisphosphonates (N-BPs). HMGCS1—hydroxymethylglutaryl-CoA synthase, HMG-CoA—3-hydroxy-3-methylglutaryl coenzyme A, HMGCR—3-hydroxy-3-methyl-glutaryl-coenzyme A reductase.

The mevalonate pathway is a key metabolic pathway that supplies isoprenoid units and plays a central role in the biosynthesis of important molecules, including cholesterol, steroid hormones, heme *a* and coenzyme Q (CoQ) (Figure 2) [25,26]. The mevalonate pathway has been the subject of in-depth research due to its importance in the pathophysiological processes of cancer, cardiovascular and neurodegenerative diseases [27,28,29].

### 2.1. Cholesterol

Cholesterol, the major sterol in mammals, is found in all cell membranes and forms lipid rafts that are an important part of signaling complexes. It constitutes about one third of lipids in the plasma membrane, packaging phospholipids into membranes and insuring their fluidity and barrier function [30]. Sterols present in cell membranes are mainly produced endogenously. When intracellular cholesterol levels decrease, sterol regulatory element-binding proteins (SREBPs), particularly SREBP-1 and SREBP-2, are activated via proteolytic cleavage and translocate to the nucleus, where they upregulate genes involved in lipid and cholesterol biosynthesis, such as HMG-CoA reductase and low-density-lipoprotein (LDL) receptor [31]. These regulatory proteins are essential for maintaining cholesterol within a physiologically safe range and ensuring its structural integrity in cellular membranes. Cholesterol contributes to membrane fluidity and stability, which is crucial for the function of membrane-bound proteins, including those involved in mitochondrial electron transport. By controlling lipid synthesis, SREBPs indirectly influence mitochondrial membrane composition and redox balance, thereby contributing to the stability of cholesterol molecules and overall mitochondrial function. Cholesterol is also essential as a precursor for the production of steroid hormones. Increasing evidence suggests that cancer cells are highly dependent on high cholesterol levels, moderating the antiviral response of type I interferon (IFN) in macrophages while avoiding detection by the immune system [32].

### 2.2. Dolichol

Dolichol, a polyprenol, is another product of the mevalonate pathway which plays an important role in protein glycosylation. As an essential oligosaccharide carrier, it is involved in the synthesis of C-mannosylation, glycosylphosphatidylinositol (GPI)-anchored protein and N-glycans [33]. It was described over 30 years ago that the level of dolichol increases with age in the human brain, although in neurodegenerative diseases such as Alzheimer’s, the situation is the opposite, showing possible increases in dolichyl phosphate glycosylation as part of the disease’s pathophysiological processes [34].

### 2.3. CoQ

**Figure 3:**
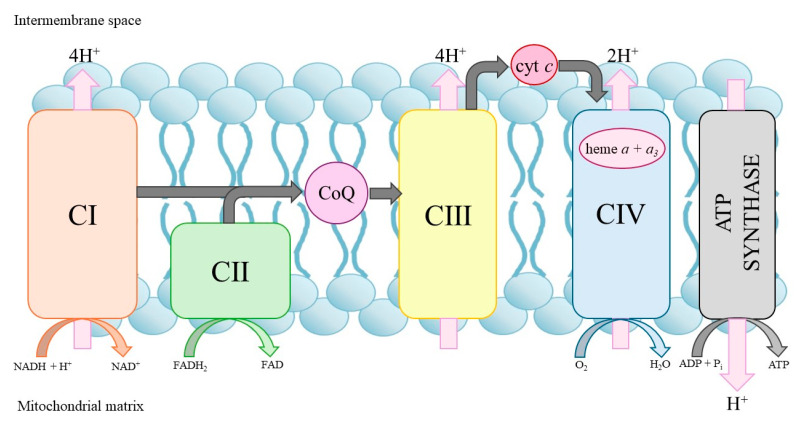
Mitochondrial oxidative phosphorylation system. cyt *c*, cytochrome *c*; CI–CIV, respiratory chain complexes.

CoQ is a lipid-soluble molecule found in cell membranes. Although the human body is able to synthesize it, it is also abundant in our diet. CoQ is an important electron transport carrier of the respiratory chain involved in producing ATP via the mitochondrial oxidative phosphorylation system (Figure 3) [35]. CoQ moves across the inner mitochondrial membrane, transporting electrons from substrate dehydrogenases (complexes I and II), where it is reduced to complex III, facilitating electron transport in the respiratory chain. In addition, CoQ is an antioxidant and can replenish the pool of other antioxidants, such as vitamin C or E, by reducing their oxidized form. The antioxidant properties of CoQ are largely dependent on processes that renew the reduced pool of CoQH_2_ [36]. However, if the molecule is not fully reduced, a semiquinone radical can be formed, which reacts with oxygen to produce a precursor of various reactive oxygen species (ROS)—the superoxide anion. CoQ levels naturally decline with age, and decreased concentrations have also been observed in various pathological conditions, including diabetes, cancer and neurodegenerative disorders [37].

### 2.4. Heme a

Heme *a* is an essential molecule in organisms that depend on aerobic respiration. It is embedded in electron transport chains as a prosthetic group in respiratory oxidases containing cytochrome *a* [38]. In mammals, hemes *a* + *a*_3_ are components of cytochrome *c* oxidase (COX, complex IV of the mitochondrial respiratory chain) (Figure 3). Mutations in the COX15 gene encoding heme *a* synthase cause fatal infantile hypertrophic cardiomyopathy [38]. On the other hand, increased levels of heme *a* were found in Alzheimer’s disease patients [39]. Furthermore, the accumulation of COX subunit 1 protein and other mitochondrial markers in autophagocytic vesicles is associated with a higher level of mitochondrial turnover.

### 2.5. Protein Prenylation

Protein prenylation is essential for cell growth, cell division, receptor trafficking and cell polarization. It is the first step in targeting and binding to the cell membrane and also mediates protein–protein interactions. Interest in the process of protein prenylation increased following the description of protein prenylation as being required for the malignant activity of oncogenic Ras proteins [40].

The two main isoprenoid molecules involved in protein prenylation, produced in the mevalonate pathway, are farnesyl diphosphate (FPP) and geranylgeranyl diphosphate (GGPP) [41]. GGPP is crucial for the membrane localization of small GTP-binding proteins such as Ras, Rho, Rac and Rap. Ras and Rho GTPases require post-translational modifications such as farnesylation or geranylgeranylation. Prenylation allows them to associate with lipid membranes and act as a molecular switch that toggles between an inactive GDP-bound state and an active GTP-bound state. N-BPs inhibit the post-translational modifications of small GTPases by inhibiting the FPP synthase [41]. The concomitant use of N-BPs and statins, other mevalonate pathway inhibitors that block 3-hydroxy-3-methylglutaryl-coenzyme A reductase (HMG-CoA reductase), has been proposed as an effective strategy for the treatment of malignant tumors due to the inhibition of FPP and GGPP biosynthesis from two points in the pathway [42]. To inhibit tumor growth and induce the apoptosis of cancer cells, a new treatment method was proposed in which zoledronate is placed in a liposome, which allows the bypassing of the high affinity of the drug for bone [43]. Interestingly, this technique led to the inhibition of FPP synthase activity and the loss of protein prenylation, and also allowed zoledronate to cross the blood–brain barrier, as detected in the brains of the tested mice. It has been suggested that prenylation inhibitors should be further investigated as a potential new class of anti-cancer drugs.

Emerging evidence suggests that geranylgeraniol (GGOH) supplementation can partially reverse the inhibition of protein prenylation induced by N-BPs by replenishing the GGPP pool in various cell types and animal models [24,44,45,46]. Given its safety profile and availability as a dietary supplement, GGOH has potential as an adjunct treatment for BRONJ [47]. GGOH has shown promise in counteracting the adverse effects of N-BPs by promoting bone remodeling, reducing tissue toxicity, and enhancing local angiogenic responses. However, further studies are needed to elucidate the optimal dose, methods of administration, and mechanisms of action in both preclinical and clinical settings.

## 3. Inhibition of the Mevalonate Pathway by N-BPs

### 3.1. Effects on the Cellular Level

#### 3.1.1. Effects on Bone Cells

The main targets of N-BPs are osteoclasts. The selective absorption of N-BP to sites with high bone resorption rates causes the drugs to come into close contact with osteoclasts, which translates into their high safety and efficacy [48]. N-BPs reduce bone resorption by inhibiting osteoclast recruitment and resorption activity and inducing apoptosis. N-BPs, by inhibiting FPP synthase, prevent protein prenylation, which is crucial for osteoclasts to obtain resorption activity. The correlation between FPP synthase inhibition and the antiresorptive activity of N-BPs was shown over 20 years ago by Dunford et al. [49].

Despite the expected effect of reducing the rate of bone resorption, N-BPs also act on other cells with which they come into contact [50]. It has been shown that these drugs can alter the expression of osteoprotegerin (OPG) and macrophage colony-stimulating factor (M-CSF) in osteoblasts, affecting osteoblastogenesis. Variable effects on the proliferation and differentiation of osteogenic cells have been observed when different concentrations of N-BPs were administered. The anti-apoptotic properties of N-BPs towards osteoblasts are observed when using concentrations ranging from 10^−6^ to 10^−8^ M. Pro-apoptotic events in osteoblasts and osteocytes are observed after increasing the dose to 10^−4^–10^−5^ M.

N-BPs inhibit bone resorption, leading to lower serum calcium levels [16,51,52]. The P-C-P moiety in N-BPs is responsible for quick binding to the bone mineral. However, the different substitutions in the R_1_ and R_2_ positions in the carbon atom allow them to chelate Ca^2+^ more effectively [53]. Most studies focus on N-BPs’ effects within osteoclasts or their precursors, particularly concerning calcium-related ion channels. For example, Sheng-Nan et al. investigated the effects of ibandronate on intermediate-conductance Ca^2+^-activated K^+^ (IK_Ca_) channels in the osteoclast precursor cell line RAW 264.7 [54]. They found that ibandronate inhibited IK_Ca_ channel activity, resulting in membrane depolarization and reduced cell migration.

#### 3.1.2. N-BP Effects on Off-Target Cells

The way in which BP treatment alters cellular processes and leads to apoptosis depends on their molecular structure [10]. Non-nitrogen containing BPs bind to ATP, leading to the production of non-hydrolyzable ATP analogs that compete with the nucleotide, resulting in impaired energy metabolism and apoptosis. N-BPs inhibit FPP synthase, a key enzyme of the mevalonate pathway, thereby preventing the prenylation and activation of small GTPases that are essential for cytoskeletal organization, vesicular trafficking, cell survival and other cellular processes. In vitro studies demonstrating the toxicity of both types of BPs (N-BPs and non-nitrogen containing BPs) do not contain information allowing a comparison of the concentrations of these drugs with the concentrations observed in the plasma of patients undergoing BP therapy. In the case of studies with N-BPs, depending on the cell line, their concentrations range from 1 µM in the human endothelial cell line (EA.hy926) to 5 µM in the human epithelial cell line (HaCaT) and even 100 µM in the umbilical vein endothelial cell line (HUVEC) [55,56,57].

##### Anti-Cancer Effects, Cytoskeleton Alterations

Relatively little is known about the effects of N-BPs on tissues other than bone. Most of the existing knowledge comes from studies of cancer cells, as N-BPs are used in anti-cancer therapies [58]. N-BPs, such as zoledronic acid, are widely used to prevent skeletal-related events in cancer patients with bone metastases. Beyond their bone-targeting properties, preclinical studies have demonstrated that N-BPs exert direct anti-cancer effects. These effects include the inhibition of tumor cell proliferation, induction of apoptosis, and modulation of the tumor microenvironment. For instance, N-BPs have been shown to stimulate the expansion and cytotoxic activity of human T cells, which possess potent anti-tumor capabilities. The anti-tumor effect of N-BPs, exerted through the blocking of the prenylation of small GTPases, has been explained by the disruption of cytoskeletal organization [59]. After adding 10^−5^ M alendronate to human prostate cancer (PC-3) cells, Virtanen at al. observed significant changes in the organization of F-actin and cofilin levels. The phosphorylation of cofilin is crucial for actin filament elongation, which is carried out by Rho GTPases. This observation led to the hypothesis that N-BPs, which cause a loss of prenylation of small GTPases, may be associated with lower levels of phosphophilin, which disrupts the balance between phosphorylated and unphosphorylated cofilin, affecting actin dynamics [60,61]. Morphological changes caused by disturbances in the polymerization of the actin cytoskeleton were observed during zoledronate treatment in fibroblasts, vascular smooth muscle cells, and tumor cells [62,63,64].

While N-BPs demonstrate promising anti-cancer properties, their off-target toxicity, particularly in renal, cardiovascular, and soft tissues, raises concerns. Innovative strategies such as nanoparticle-mediated delivery, molecular docking, tumor-targeted conjugates, and controlled-release formulations offer potential for increased specificity and safety [65,66,67,68,69,70]. A careful balance between risks and benefits, supported by individual patient assessment, especially those with renal impairment, vitamin D deficiency, or pre-existing hypocalcemia, is essential to maximize therapeutic impact while minimizing adverse effects.

##### Effects Related to Nephrotoxicity

In human embryonic kidney (HEK-293) cells, zolendronate increases ROS production and induces oxidative damage, as evidenced by the altered expression of genes related to oxidative stress and apoptosis [71]. Additionally, endoplasmic reticulum (ER) stress-mediated apoptosis has been observed. Studies in zoledronate-treated rats and renal tubular HK-2 cells suggest that zoledronate-induced nephrotoxicity is linked to disruptions in glutathione biosynthesis and the tricarboxylic acid (TCA) cycle, leading to excessive ROS production, oxidative stress, and inflammation, thereby leading to nephrotoxicity [72].

##### Effects on Endothelial Cell Viability, Inflammation, and Apoptosis

N-BPs have been shown in vitro to reduce the viability of human endothelial cells, induce their inflammatory response and impair the endothelial differentiation potential of human mesenchymal stem cells [57,73,74]. One of the signaling pathways involved in endothelial cell proliferation, apoptosis, and angiogenesis is the EGFR/Akt/PI3K pathway, which begins with the activation of the epidermal growth factor receptor (EGFR) by ligands, which then triggers the activation of phosphatidylinositol 3-kinase (PI3K) and serine/threonine kinase (Akt), releasing the Bcl2-related cell death antagonist [75]. The Akt pathway is responsible for the activation of nuclear factor κB (NFκB), reprogramming the expression of genes whose products are involved in stress responses, inflammation, and the inhibition of apoptosis. N-BPs are considered good candidates for anti-cancer activity because they inhibit the key cell survival pathway by inhibiting the activation of Akt and NFκB [76]. Moreover, zoledronate has been shown to impair endothelial cell function and survival by inhibiting the extracellular signal-regulated protein kinase 1/2 (ERK1/2) pathway, which is another prenylation-dependent signaling cascade [77]. Importantly, ERK1/2 also serves as an anti-inflammatory signal to prevent inflammatory signaling in endothelial cells [78]. Endothelial cells treated with zoledronate but not alendronate exhibited a significant decrease in active phospho-ERK1/2 levels, along with increased expression of inflammatory markers [intercellular adhesion molecule 1 (ICAM1) and cytokine interleukin-6 (IL6)], indicating endothelial cell activation and the initiation of local inflammation through the upregulation of inflammatory cytokines and adhesion molecules [57]. In osteoblasts and fibroblasts, zoledronate, unlike alendronate, also induced an increased level of inflammatory cytokines [79]. These observations indicate that zoledronate induces a more potent inflammatory response than alendronate, although both N-BPs have a similar effect on ERK1/2 phosphorylation [57].

##### Effects on Endothelial Cell CoQ Homeostasis and Energy Metabolism

N-BPs, by inhibiting the mevalonate pathway, lead to the inhibition of synthesis of CoQ, an important intracellular antioxidant that is present in all cell membrane [80,81,82]. Reduced CoQ (CoQH_2_), by binding free radicals, inhibits lipid peroxidation processes and prevents oxidative modifications of DNA and proteins. Decreased CoQ levels due to age or blockade of the mevalonate pathway by drugs (e.g., statins) may result in oxidative stress and damage. For example, patients with coronary heart disease have lower levels of plasma CoQ, which contributes to lipid peroxidation and DNA damage [83]. In 2014, for the first time, an (for a long time, the only) association was observed between N-BP therapy and reduced levels of CoQ10 in the plasma of postmenopausal women [84]. However, currently, CoQ10 supplementation is rarely recommended [84,85]. Reductions in CoQ levels in tissues or organs during N-BP treatment have not yet been investigated, apart from our findings in endothelial cells [57,85]. We recently showed that both alendronate and zoledronate significantly reduce CoQ levels in EA.hy926 human endothelial cells [57]. In particular, in vitro treatment with 2.5 µM zoledronate caused a 60% decrease in cellular CoQ content. N-BPs disrupted cellular CoQ redox homeostasis by decreasing the CoQ redox state (CoQH_2_/CoQ). The significant N-BP-induced CoQ deficiency seems to contribute to metabolic reprogramming, including reduced mitochondrial respiratory function and reduced ATP production. A general decline in mitochondrial respiration has been observed in N-BP-treated endothelial cells, particularly with stronger reducing substrates such as pyruvate and glutamate. However, oxidative capacity as well as aerobic metabolism in the N-BP-treated endothelial cells were increased. Impairment of the oxidative phosphorylation system in N-BP-treated endothelial cells was evidenced by reduced ATP-linked respiration with stronger reducing substrates and decreased cellular ATP levels. These alterations in oxidative metabolism resulted in elevated intracellular oxygen levels. N-BP-treated endothelial cells showed significant increases in histone lysine-specific demethylase 6A (KDM6A), a direct oxygen sensor involved in the regulation of gene transcription, along with significant reductions in hypoxia-inducible factor 1α (HIF1α), a key marker of cellular hypoxia. These observations suggest the presence of elevated oxygen levels in N-BP-treated cells, which may enhance ROS production and contribute to oxidative stress. In N-BP-treated endothelial cells, a decrease in CoQ level, particularly its antioxidant-active reduced form, was accompanied by increased total and mitochondrial ROS production, resulting in oxidative stress, as evidenced by elevated levels of the antioxidant enzymes glutathione reductase and superoxide dismutase (SOD1). These studies represent the first comprehensive investigation into the effects of N-BPs on cellular energy metabolism, using endothelial cells as a model system.

##### Effects on Lipid Metabolism

An increase in palmitate oxidation was observed in EA.hy926 endothelial cells treated with zoledronate or alendronate, indicating a metabolic shift towards fatty acid catabolism [57]. In vivo, zoledronate treatment reduced hepatic lipid accumulation in mice fed a high-fat diet, suggesting a potential regulatory effect on lipid metabolism in liver cells [86]. Zoledronate deceased the expression of genes involved in fatty acid oxidation and reduced both free fatty acid levels and lipid droplet size in the hepatocytes of treated mice. It has been proposed that zoledronate suppresses de novo lipogenesis regulated by sterol regulatory element-binding transcription factor 1c (SREBP-1c) by inhibiting RhoA GTPase activation, a process linked to reduced levels of FPP and GGPP [86]. In contrast to these findings, Cheng et al. reported that zoledronate decreased fatty acid oxidation and promoted their accumulation in human kidney (HK-2) cells [87]. It has been proposed that although the β-oxidation of fatty acids is the primary energy source for renal tubular epithelial cells, zoledronate-induced excessive fatty acid accumulation and renal fibrosis, mediated by the transforming growth factor β (TGFβ) signaling pathway, are major contributors to its nephrotoxicity. Nonetheless, the potential involvement of impaired protein prenylation and dysregulated GTPase activity in zoledronate-induced renal toxicity cannot be excluded [87]. The cell type-specific effects of zoledronate on lipid metabolism may be attributed to differences in metabolic signaling pathways, metabolic substrate preferences, mitochondrial function or mevalonate pathway activity. These discrepant responses highlight the importance of tissue context in interpreting the metabolic effects of N-BPs and indicate the need for further studies involving other tissues and cell types.

##### Effects on Autophagy, Mitochondrial Dynamics, and Turnover

Defective autophagy has also been linked to a severe reduction in protein prenylation, which may lead to inflammasome activation and subsequent cell death [88]. However, the effects of N-BPs, as inhibitors of the mevalonate pathway, on autophagy in non-bone cells remain largely unexplored.

The mevalonate pathway supports the prenylation of small GTPases, including Rab proteins, which are essential for mitochondrial quality control processes such as fission and mitophagy [88,89,90]. However, this aspect remains poorly investigated in the context of N-BP inhibition of the pathway, and so far, only one study has shown that N-BPs can affect mitochondrial dynamics and turnover [57]. In EA.hy926 human endothelial cells treated with N-BPs, no changes were observed in the levels of mitochondrial biogenesis markers, including peroxisome proliferator-activated receptor γ coactivator 1α (PGC1α) and nuclear factor erythroid 2-related factor 2 (NRF2) [57]. However, the reduction in mitochondrial fission markers—mitochondrial fission factor (MFF) and active phospho-dynamin-related protein 1 (phospho-DRP1)—along with unchanged levels of the fusion marker optic atrophy protein 1 (OPA1) suggests reduced mitochondrial clearance via mitophagy in N-BP-treated endothelial cells, likely as a consequence of ERK1/2 signaling downregulation. This study provides the first evidence that N-BPs alter mitochondrial dynamics and turnover. Mitochondrial fission is a prerequisite for mitophagy, the selective degradation of damaged mitochondria via autophagy [91]. Fission facilitates the segregation of damaged mitochondrial components into smaller fragments that are more readily engulfed by autophagosomes. Reduced expression or activity of key proteins involved in fission (including Drp1) has been linked to impaired mitophagy, leading to the accumulation of dysfunctional mitochondria [92,93,94]. Disrupted mitochondrial dynamics and mitophagy can lead to various pathologies, including neurodegenerative disorders like Parkinson’s and Alzheimer’s, cardiovascular and metabolic disorders, and cancer [91]. Therefore, investigating the consequences of N-BP-induced changes in mitochondrial dynamics and quality control mechanisms is crucial to deepen our understanding of their biological impact.

##### Effects on Calcium Homeostasis

N-BPs can lower plasma calcium levels by inhibiting bone resorption, thereby reducing the release of calcium into the bloodstream [16,51,52]. Currently, there is limited evidence that N-BPs disrupt calcium homeostasis in tissues beyond bone and blood. For example alendronate has been shown to affect cellular calcium dynamics and signaling in cardiomyocytes [95] and chondrocytes [96] in vitro, potentially altering contractile or metabolic function. Additionally, transcriptomic analyses in cancer patients have revealed changes in calcium signaling pathways in regulatory T cells [97], suggesting potential immunometabolic effects. Although these findings are limited, they suggest the possibility of tissue-specific effects of N-BPs on calcium-dependent processes and warrant further investigation. It is important to note that these local cellular effects may, in part, be secondary to the systemic hypocalcemia induced by N-BP administration.

### 3.2. N-BP Effects on the Mitochondrial Level

Mitochondria are the site of oxidative phosphorylation, the process by which cells produce ATP from nutrients [98,99]. Oxidative phosphorylation occurs across the inner mitochondrial membrane via the electron transport chain and ATP synthase (Figure 3). Mitochondria are also key guardians of cellular homeostasis. In addition to synthesizing ATP via oxidative phosphorylation, mitochondria are also major sources of ROS. While ROS at the physiological level function as critical signaling molecules involved in maintaining cellular homeostasis, excessive ROS production becomes detrimental. When the balance is disturbed, elevated levels of ROS can damage lipids, proteins, and DNA, contributing to oxidative stress and playing a central role in the pathogenesis of various diseases, including neurodegeneration, cancer, and cardiovascular disorders. In addition, impaired mitochondria release apoptotic factors that act as signals that induce cell death. Mitochondria also play an important role in Ca^2+^ homeostasis by buffering intracellular calcium levels, thereby supporting proper cell function and survival [100].

#### 3.2.1. Effects on Mitochondria-Induced Apoptosis

Mitrofan et al. investigated the mechanism of zoledronate-induced apoptosis in the HF28RA human follicular lymphoma cells and identified key mitochondrial events involved in this process [101]. Zoledronate treatment led to mitochondrial membrane permeabilization, which resulted in the release of cytochrome *c* into the cytosol, activation of caspase-3, and DNA fragmentation, i.e., hallmarks of mitochondrial-dependent apoptosis. The study identified inhibition of the mevalonate pathway as a major trigger of this response. Geranylgeraniol (GGOH) supplementation, which restores the prenylation of proteins downstream of the blocked pathway, effectively rescued cells from zoledronate-induced apoptosis, underscoring the importance of prenylated proteins in maintaining mitochondrial integrity and cell survival. Thus, N-BPs can activate the intrinsic pathway of apoptosis by interfering with mitochondrial function through the loss of membrane potential, oxidative stress, and impaired protein prenylation. Although the exact molecular link between N-BP and mitochondrial apoptosis signaling remains unclear, the results support potential therapeutic strategies combining N-BPs with agents that modulate mitochondrial sensitivity or protein prenylation to enhance their efficacy, particularly in resistant cancer cells.

#### 3.2.2. Effects on Mitochondrial Calcium Homeostasis

Some evidence suggests that N-BPs may influence mitochondrial calcium handling in kidney cells. For instance, studies have reported reduced calcium release from renal mitochondria in vitro [102] as well as increased mitochondrial calcium levels in vivo [103]. Interestingly, etidronate has been shown to protect the kidney from ischemic injury [104]. This protective effect is proposed to result from enhanced mitochondrial calcium sequestration, which helps prevent intracellular Ca^2+^ overload.

#### 3.2.3. N-BP-Induced Mitochondrial Heme a Decrease

Mevalonic acid serves as a precursor for the synthesis of isoprenoid unit-containing heme *a*, an essential prosthetic group of cytochrome *c* oxidase (complex IV) [105]. In EA.hy926 endothelial cells, treatment with inhibitors of the mevalonate pathway, zoledronate or alendronate, led to a reduction in cytochromes *a* + *a*_3_, suggesting a reduction in the heme *a* content of complex IV [85]. Despite this reduction, the maximum enzymatic activity of complex IV in intact endothelial mitochondria remained unchanged compared with control untreated cells. Interestingly, this is the only study to date to directly examine the effect of N-BPs on mitochondrial heme *a* levels, emphasizing the need for further studies to elucidate the broader implications of N-BP-induced disruption of mitochondrial heme *a* biosynthesis and respiratory chain integrity.

#### 3.2.4. N-BP-Induced Changes in Mitochondrial Respiratory Function

Mitochondrial dysfunction induced by N-BPs remains an underexplored area and relatively few studies have addressed this aspect. In mitochondria isolated from the kidneys of zoledronate-treated rats, significant decreases in mitochondrial dehydrogenase activities, mitochondrial membrane depolarization, and permeabilization as well as ATP depletion were observed [106]. In mitochondria isolated from endothelial cells treated with alendronate or zoledronate, an overall reduction in the oxidation of respiratory substrates was observed, with the exception of increased fatty acid oxidation [85]. These mitochondria exhibited reduced respiratory rates, diminished membrane potential, and lower efficiency of ATP synthesis during the oxidation of complex I and II substrates. Moreover, in the mitochondria of endothelial cells treated with N-BPs, a reorganization of respiratory chain supercomplexes was observed, characterized by a downregulation of the complex III_2_ + IV supercomplex and the complex III_2_ dimer, while the protein level of complex III and the activity of complex I within CI + CIII-related supercomplexes (I + III_2_ + IV_(n)_ and I + III_2_) remained unchanged. These changes were accompanied by reduced protein levels and enzymatic activities of complexes II and III, as well as ATP synthase, indicating impaired structural and functional integrity of the oxidative phosphorylation system. The exact mechanism by which N-BPs induce these changes is not yet known; however, it is likely that this molecular reorganization helps optimize the utilization of deficient mitochondrial coenzyme Q (mtCoQ) (see below) and maintain electron flow through the complex I-III-IV pathway [85]. The observed decrease in ATP synthase protein levels and activity suggests a corresponding reduction in ATP synthesis.

#### 3.2.5. N-BP-Induced mtCoQ Deficiency

N-BPs as inhibitors of the mevalonate pathway may disturb the synthesis of isoprenoid-derived molecules, including mtCoQ, a key electron carrier in the mitochondrial electron transport chain (Figure 3) [81,82]. Reduced mtCoQ (CoQH_2_) participates in the production of mitochondrial ROS through the formation of superoxide/H_2_O_2_ due to electron leakage from the semiubiquinone radical at the mtCoQ-biding sites of the respiratory chain. mtCoQ redox homeostasis, maintained through its cycling between oxidized and reduced forms, is crucial for efficient electron transport and the regulation of oxidative stress [81].

Recently, we demonstrated for the first time that treatment with N-BPs (alendronate or zoledronate) leads to a significant reduction in mtCoQ level. In EA.hy926 endothelial cells treated with alendronate or zoledronate, a 45–55% reduction in total mtCoQ was observed [85]. Importantly, the pool of reduced mtCoQ (mtCoQH_2_), which is essential for its antioxidant function, was lost. The disrupted mtCoQ redox homeostasis induced a compensatory response characterized by increased expression of mitochondrial antioxidant proteins, including superoxide dismutase 2 (SOD2) and uncoupling protein 2 (UCP2). Moreover, N-BP treatment resulted in a significantly increase in mitochondrial H_2_O_2_ production, which has been attributed to increased mtCoQ reduction level (elevated mtCoQH_2_/mtCoQtot ratio). These findings suggest that decreased mitochondrial CoQ levels, due to mevalonate pathway inhibition by N-BPs, may impair mitochondrial respiratory function and disrupt mtCoQ redox balance, leading to elevated mitochondrial ROS production. These results underscore the need for further studies on mtCoQ deficiency induced by N-BP treatment. Severe mtCoQ depletion, coupled with excessive mitochondrial ROS production, can disrupt mitochondrial integrity and cellular homeostasis. Such dysfunctions are known to contribute to the pathogenesis of several diseases, including cardiovascular and neurodegenerative diseases, and may be associated with accelerated aging and reduced lifespan [107]. Elucidation of the mechanisms underlying mtCoQ depletion and its downstream effects may point to new strategies to alleviate N-BP-induced mitochondrial toxicity, particularly through CoQ10 supplementation, which may help restore mitochondrial function and redox balance.

#### 3.2.6. Potency and Mitochondrial Effects of Second-Generation and Third-Generation N-BPs

The effects of N-BPs on mitochondria described so far do not indicate specific differences in mitochondrial toxicity between second-generation and third-generation N-BPs. To our knowledge, only one study has directly compared their effects on mitochondrial function in endothelial cells, using 5 μM of alendronate (second-generation) and 1 μM of zoledronate (third-generation) [85]. This study reported similar qualitative effects on mitochondrial function for both N-BPs, despite a lower concentration of zoledronate, suggesting greater potency rather than a mechanistic difference. Therefore, while this limited evidence suggests comparable mitochondrial outcomes, the differential potency of third-generation N-BPs warrants further investigation to determine whether there are subtle or long-term toxicological differences. Moreover, the increased efficacy of third-generation N-BPs may impact the occurrence of mitochondrial side effects, justifying the need for further studies to ensure safer clinical use.

## 4. Conclusions

This is the first review to comprehensively synthesize current knowledge on the mitochondrial effects of N-BPs in non-skeletal cells. By consolidating evidence from cellular and molecular studies, this review highlights mitochondrial dysfunction as a potential central mechanism underlying a wide range of off-target effects. This new perspective brings attention to a largely underexplored aspect of N-BP pharmacology and aims to support future research directions and therapeutic considerations that incorporate mitochondrial health into N-BP safety assessments.

Although widely used N-BPs are clinically effective in treating bone resorption disorders, they are increasingly recognized as drugs with a broad spectrum of adverse effects resulting from inhibition of the mevalonate pathway in non-skeletal cells. The cellular consequences of N-BP exposure include anti-tumor activity, cytoskeletal disruption, renal toxicity, endothelial dysfunction, and alterations in lipid and energy metabolism. Many of these effects may be related to mitochondrial dysfunction, including impaired respiratory function, impaired calcium homeostasis, deficiencies in CoQ and heme *a* in mitochondria and activation of mitochondria-dependent apoptosis. These mitochondrial disturbances can significantly impact cellular energy metabolism and viability. However, at this stage, specific biomarkers indicative of mitochondrial dysfunction in non-skeletal tissues suitable for clinical monitoring after N-BP treatment have yet to be identified.

Despite the growing awareness of the off-target effects, the impact of N-BPs at the mitochondrial level remains a largely unexplored area of research. In particular, the role of mitochondrial CoQ deficiency as a contributor to N-BP toxicity is understudied, and the potential for CoQ supplementation to ameliorate these effects has not been investigated. To date, no preclinical (cell or animal models) or clinical studies have investigated CoQ10 supplementation in the context of N-BP therapy or its potential to alleviate N-BP-induced mitochondrial dysfunction. In various clinical contexts unrelated to N-BP therapy, CoQ10 supplementation—typically at doses of 100–300 mg daily for several weeks or months—has been shown to be effective in improving mitochondrial function and reducing oxidative stress [108].

## 5. Future Perspectives

Future perspectives on N-BP-induced mitochondrial dysfunction should be expanded beyond current in vitro systems to include diverse cellular models and in vivo animal models, enabling comprehensive analysis of mitochondrial parameters that may be affected by inhibition of the mevalonate pathway, as discussed in this review. They should include an in-depth exploration of N-BP-induced mitochondrial dysfunction, with a focus on respiratory chain function and mitochondrial quality control mechanisms. It is also important to identify reliable mitochondrial biomarkers that can enable the early detection of off-target toxicity in non-skeletal tissues. Furthermore, studies should investigate whether the mitochondrial effects of N-BPs differ between different generations of these compounds, which may have implications for drug selection and safety. Particular attention should be paid to the evaluation of CoQ supplementation as a potential protective strategy to ameliorate mitochondrial impairment and disruption of cellular energy metabolism. Although the efficacy of antioxidant therapies in the context of N-BP-induced mitochondrial toxicity has not been studied so far, other antioxidants acting on mitochondria, such as MitoQ, which accumulates in mitochondria and directly neutralizes ROS, may also represent potential therapeutic agents beyond the use of CoQ10 to alleviate oxidative stress and preserve mitochondrial function. Further studies on the complex interplay between the mevalonate pathway, mitochondrial function, and overall cellular homeostasis will be important to optimize the clinical use of N-BPs while reducing unintended effects on non-target tissues.

## Figures and Tables

**Table 1:** Documented major side effects of BP therapy [22].

Side Effects	BPs
Osteomalacia	Etidronate
Hypocalcemia	Zoledronate, pamidronate, clodronate
Acute phase reaction	Zoledronate, ibandronate
Gastrointestinal side effects	Orally administered N-BPs, alendronate
Nephrotoxicity, renal failure	Zoledronate, pamidronate, ibandronate, alendronate
Ocular side effects	Pamidronate, zoledronate
Bisphosphonate-related osteonecrosis of the jaw (BRONJ)	Zoledronate
